# Development and Characterization of Sustained-Released Donepezil Hydrochloride Solid Dispersions Using Hot Melt Extrusion Technology

**DOI:** 10.3390/pharmaceutics13020213

**Published:** 2021-02-04

**Authors:** Abdullah Alshetaili, Bjad K. Almutairy, Sultan M. Alshehri, Michael A. Repka

**Affiliations:** 1 Department of Pharmaceutics, College of Pharmacy, Prince Sattam Bin Abdulaziz University, Al-Kharj 11942, Saudi Arabia; b.almutairy@psau.edu.sa; 2 Department of Pharmaceutics, College of Pharmacy, King Saud University, Riyadh 11451, Saudi Arabia; salshehri1@ksu.edu.sa; 3 Department of Pharmaceutical Sciences, College of Pharmacy, Almaarefa University, Riyadh 11597, Saudi Arabia; 4 Department of Pharmaceutics and Drug Delivery, School of Pharmacy, The University of Mississippi, Oxford, MS 38677, USA; marepka@olemiss.edu

**Keywords:** donepezil hydrochloride, hot-melt extrusion, hydrophilic carriers, solid dispersion, sustained release

## Abstract

The aim of this work was to develop the sustained release formulation of donepezil hydrochloride (DH) using the hot-melt extruded solid dispersion technique via the rational screening of hydrophobic carriers. Hydrophobic carriers with different physicochemical properties such as pH-independent swellability, low-permeability (Eudragit^®^ RS PO (E-RS)), pH-independent non-swellability (ethyl cellulose N7 (EC-N7)), and the presence of lipids (Compritol^®^ 888 ATO (C-888)) with or without pore-forming agents were used to achieve the sustained release profile of DH. Mannitol (MNT) was chosen as the temporary pore-forming agent. The thermal analysis showed that both the drug and C-888 preserved their crystallinity within a solid dispersion. During a dissolution test, MNT could generate pores, and the drug release rate was proportionally correlated to the MNT content. Tailoring of the ratio of C-888 and MNT in the formulations along with an appropriate extrusion temperature profile resulted in the modified release of DH, and a preferable release pattern was obtained under these conditions. C-888 was chosen for the further investigations to obtain tablets with a high integrity. The optimized tablets were compared to the marketed formulation of Aricept^®^ in terms of drug release profiles. The optimized formulation showed the stable and sustained release behavior of extended release profile, which was close to the release behavior of Aricept^®^ with good tablet characteristics. It was concluded that the hot-melt extrusion technique can be utilized for the manufacturing of DH sustained release tablets with improved tablet integrity and characteristics by co-processing the tablet excipient with DH/C-888.

## 1. Introduction

Donepezil hydrochloride (DH) (melting point: 220–222 °C) is a reversible inhibitor of acetylcholinesterase. It produces its therapeutic effects by increasing the concentration of acetylcholine and enhancing the cholinergic function in the brain. It is used for the treatment of mild-to-moderate dementia associated with Alzheimer’s disease [1]. The aqueous solubility of DH is ≥20 mg/mL. Therefore, it is considered to be a highly water-soluble active pharmaceutical ingredient (API). Ethyl cellulose N7 (EC-N7) is a non-swellable cellulose ethyl ether and an insoluble polymeric carrier. Cationic Eudragit^®^ RS PO (E-RS) is another example of a hydrophobic/insoluble carrier [2]. The lipid matrix of Compritol^®^ 888 ATO (glyceryl behenate United States Pharmacopoeia-National Formulary [USP-NF]) (C-888) is a pH-independent carrier. It has a melting point of around 74 °C and a hydrophile–lipophile balance (HLB) of 2. All these hydrophobic carriers including EC-N7, E-RS, and C-888 are used to extend the release of water-soluble APIs [2,3,4].

Hot-melt extrusion (HME) is a common technology that can be applied to several pharmaceutical preparations, especially to solid dispersions. There are some parameters of HME that should be considered, such as the feeding rate, shearing force, temperature, die geometry, barrel design, and screw rotating speed. With the optimization of these parameters, the formulators can contribute to the production of a desired final product with a preferable drug release profile and a uniformity of size, shape, and drug content. HME offers some advantages over other conventional methods. For example, it is a one-step, solvent-free process that has a continuous operation and a scalable process that has fewer processing steps [5]. In addition, it requires no compression, and it can improve bioavailability due to the dispersion of the drug at the molecular level in such a dosage form [5,6,7]. The growing demand for versatile continuous processes in the pharmaceutical industry has made HME a preferable technique in their production lines. The reductions of waste, energy, and costs are the advantages of continuous process compared to a single batch process such as heat fusion (melt and mix methods) [8,9,10]. Some conventional methods like sintering techniques have been investigated [11]. Therefore, these techniques could mimic the residence time when the screw speed of the extruder is decreased. The main limitation of HME in the pharmaceutical industry is that a higher energy input is required to produce products compared to other techniques and may exclude some thermolabile compounds due to high processing temperatures. However, in the case of lipid matrices such as C-888, there is no need for high-energy input since most of them have relatively lower melting points compared to other hydrophobic carriers such as EC-N7 and E-RS. It is widely accepted that HME technology is an innovative and feasible approach in the preparation of various pharmaceutical systems such as mini tablets, granules, immediate and modified release tablets, oral fast-dissolving systems, and transdermal drug delivery systems [6,7]. HME technology can also be used for the taste masking of bitter APIs by incorporating them into hydrophobic matrices [5,6,7].

Formulations factors that are based on different ratios of API/hydrophobic carriers and mixtures of two hydrophobic carriers with the API and with or without a pore-former, e.g., mannitol (MNT), can play a critical role in the final product characteristics. MNT can be used to produce different extended release formulations, as well as to produce different physicochemical characteristics for highly soluble drugs like DH. Controlling either formulation parameters (e.g., the ratio of DH/carrier and formulation composition) or processing parameters (e.g., processing temperature and screw speed) could play a significant role on the behavior of the drug release profile, as well as physicochemical properties of the drug within the matrix. The impact of fillers on drug content and the effects of the processing temperatures, formulation composition, tablet disintegration, DH/carrier ratio, and compression force have not been investigated yet. In a previous study, the influence of some of these parameters on the release behavior of a different API (diclofenac sodium) was studied [4,12].

Lipid aging could lead to instability during storage. It is the main disadvantage when using lipids in pharmaceutical formulations, as their physical properties could change during storage. Some of these effects are an increase of melting ranges, melting enthalpy, pore formation on the surface, rheological changes, and a decrease in tensile strength [13].

The main objectives of this study were to develop and evaluate the sustained-release DH formulations using hydrophobic carriers such as C-888, EC-N7, and E-RS while utilizing HME technology in order to avoid the side effects of DH (nightmare, insomnia, anxiety, nausea, emesis and/or diarrhea) associated with an initial sharp spike in blood DH levels for immediate release formulations. Formulations that provide the immediate release of DH are administered once a day. However, the peak plasma concentrations are reached in 2–5 h, resulting in an initial sharp spike in blood plasma levels [14]. This sharp spike could cause undesirable cholinergic side effects, as mentioned above [1,14]. In addition, the specific aims of this study were to study the effect of the extrusion temperature on a drug’s physical state and drug release profile from the lipid carrier and compare it to a conventionally processed formulation. The extrusion of pre-mixed tablet excipients (i.e., microcrystalline cellulose (MCC) and MNT) was considered to be a critical aspect in this study. Co-processing tablet excipients is expected to be a cost-effective process since there are fewer manufacturing steps, e.g., the blending of the tablet’s excipients. However, it is mandatory to investigate the effect of the added excipients on tablet’s physico-mechanical properties (e.g., drug content and tablet content uniformity, hardness, friability, and dissolution profile). To the best of our knowledge, there has been no published work on DH via HME technology.

## 2. Materials and Methods

### 2.1. Materials

DH USP was purchased from Wuhan Hengheda Pharm Co. (Wuhan, China). C-888 was kindly given by Gattefossé (Saint-Priest, Lyon, France). EC-N7 was obtained from Ashland (Ashland Aqualon Functional Ingredients, Wilmington, DE, USA). E-RS was kindly gifted by Evonik (Parsippany, NJ, USA), and Avicel^®^ PH 102 (MCC) was received from FMC Biopolymers (1735 Market Street, Philadelphia, PA, USA). Magnesium stearate was purchased from Spectrum Chemicals (Gardena, CA, USA). Pearlitol^®^ (MNT) was received as a gift sample from Roquette America Inc. (Keokuk, IA, USA). Stearic acid was purchased from Mallinckrodt Chemical Ltd. (Bedminster Township, NJ, USA). Marketed Aricept^®^ tablets were purchased from a local university pharmacy shop (Oxford, MS, USA). All chemicals and solvents utilized for analysis in the study were of analytical grade and were obtained either from Spectrum Chemicals (Gardena, CA, USA) or Thermo Fisher Scientific (Waltham, MA, USA).

### 2.2. HPLC Method for Analysis of DH

An in-house developed, reversed-phase HPLC-based analytical method was used for the determination and quantification of DH in all in vitro samples. This method was validated according to a previous research group [15]. The HPLC equipped with a UV detector, a Waters Symmetry shield, a C18 column (250 × 4.6 mm, 5 µm particle size), and an isocratic mode of elution. The mobile phase was a mixture of methanol, a 0.02 M phosphate buffer, and trimethylamine (50:50:0.5, *v*/*v*/*v*). The phosphate buffer was prepared by dissolving 2.4 g of monobasic sodium phosphate in 900 mL of water, mixing with 10 mL of trimethylamine, and adjusting to pH 2.7 ± 0.5 with phosphoric acid. The mobile phase was filtered through a nylon membrane (pore size: 0.45 µm) and degassed before use. Chromatography was performed at room temperature. The flow rate and run time for analysis were 1.0 mL/min and 15 min, respectively. In these conditions, the DH retention time (t_R_) was around 9 min. The injection volume was 20 µL, and ultraviolet detection was at a wavelength (λ_max_) of 268 nm. The column temperature was 40 °C. Finally, the acquired data were processed using the Empower 2 build 2154 software (Waters Inc., Mount Holly, NJ, USA).

### 2.3. HME Processing Method

For the screening purposes, three different carriers (C-888, EC-N7, and E-RS) were used (alone or in combination) at different ratios and processing parameters (Table 1, Table 2 and Table 3) to evaluate their effect on sustaining of the DH release. The materials were blended using a V-shell blender (GlobePharma, New Brunswick, NJ, USA, Maxiblend^®^) with and without MNT as a pore-former, and samples were analyzed for drug content and content uniformity.

Prior to HME, the materials were sieved using a size #35 USP mesh to remove any aggregated and agglomerated particles. The blended materials were extruded on a 16 mm rotating twin-screw extruder (ThermoFisher Scientific Co., Waltham, MA, USA) utilizing a round-shaped die to produce rod extrudates (EXTs). In addition, to study the effect of processing temperature on the lipid, the materials were run through the heated barrel with a screw rotating speed of 50 rpm, and the temperature for all zones of the extruder was set at 70, 75, or 77 °C except for the last zones and the die, which were set at 70 °C to cool down and solidify the EXTs (Table 4). The reason different temperatures were set was to study the effect of temperature on the drug, lipid crystallinity, and drug release profile. The processing temperature profile and screw speed were based on the physical properties of hydrophobic carriers in the preliminary studies. The resulting EXTs were further processed using a comminuting mill (Fitzpatrick, Model “L1A”) and then sieved using a size #35 USP mesh.

Another part of this study was to improve the drug content and content uniformity of the optimized formulation from the screening stage based on the drug release profile, which is referred to as DH:lipid. As shown in Table 5 and Table 6, DH (33.3–50% *w*/*w*) (1:2 and 1:1 ratios) and C-888 were blended and extruded using the same procedure and processing conditions, as previously mentioned during the screening stage but with and without premixed MCC as a tablet diluent while extruding the blend to observe the effect of the pre-mixed MCC on drug content, content uniformity, and release profile.

### 2.4. Conventional Heat Fusion Processing Method

The conventional heat fusion processing method is also known as the melt and mix method. This method was used for C-888 to compare its performance with the formulation from HME. In this processing method, C-888 was melted in porcelain casseroles at 100 °C until a homogeneous melt was obtained, and then DH was added and manually mixed to the homogenous melt. The mixture was kept to gradually cool down with continuous stirring until a congealed mass was obtained. Then, the mass melt was milled using the same milling method as mentioned before in HME-processed formulations. Subsequently, the mass was grounded, pulverized, and sieved using a size #35 USP mesh. All processed powders were stored in a desiccator at room temperature till further use.

### 2.5. TGA and Differential Scanning Calorimetry (DSC) Analysis

The thermal stability of DH and the carriers at the employed processing temperatures was determined by TGA analysis (TGA, Pyris 1 TGA Perkin Elmer, Shelton, CT, USA). A Perkin Elmer Pyris 1 TGA-running Pyris manager software (PerkinElmer Life and Analytical Sciences, Shelton, CT, USA) was utilized for TGA analysis. Drugs and excipients were evaluated for thermal stability at high temperatures. Around 3–4 mg of the sample were weighed and heated from 25 to 250 °C at a 10 °C/min heating rate under an atmosphere of nitrogen, and the TGA spectra for each sample were recorded.

Differential scanning calorimetry (DSC) (DSC, Diamond DSC, Perkin Elmer, Shelton, CT, USA) was utilized to determine the physical states of different API/carrier ratios in each physical mixture and EXT. The physical states of the pure drug, the pure carrier, physical mixtures, and EXTs were studied. The heating range used was from 30 to 250 °C at a heating rate of 20 °C/min under a nitrogen atmosphere. Due to the low melting point of the lipid, it was heated from 30 to 100 °C at the same scanning rate. The accurately weighed amount (4–5 mg) of each sample was taken in an aluminum pan and hermetically sealed. The Pyris manager software (Shelton, CT, USA) was utilized for the data processing and analysis. It was used to analyze the generated data to evaluate the crystallinity. The DSC was calibrated initially with an indium reference. In addition, the drug/lipid miscibility was estimated using the Hoftyzer and Krevelen approaches and the Hansen solubility parameters [16].

### 2.6. SEM

The surface morphology of the pure drug and milled EXTs was studied using SEM. The samples were mounted on adhesive carbon tapes placed on aluminum stubs. Gold was used to coat the samples by a Hummer^®^ 6.2 sputtering system (Anatech LTD, Springfield, VA, USA) in a high vacuum evaporator. A scanning electron microscope operating at an accelerating voltage of 0.8 kV was used for imaging (JEOL, JSM-5600, Tokyo, Japan). The morphology of formulations might have reflected the crystallinity status of the DH.

### 2.7. Solubility Parameter Calculation for Optimized Formulation

Solubility/miscibility is an important parameter in solid dispersion formulations. The Hansen solubility parameters can be obtained from the literature. However, they can also be calculated through Molecular Modeling Pro, v6.2.8 (ChemSW, Fairfield, CA, USA). This software can be conveniently used to calculate the solubility parameters for certain pharmaceutical materials by inputting their corresponding melting points and molecular structures.

### 2.8. Physico-Mechanical Evaluation for Milled EXTs for Optimized Lipid Formulation

The bulk and tapped density was calculated by measuring the volume of a 5 g milled EXT in a 10 mL graduated cylinder [17]. Firstly, the initial occupied bulk volume in the cylinder was noted. Secondly, after tapping the cylinder 100 times, the tap volume was noted. The bulk density (ρ bulk) and tap density (ρ tap) were calculated by dividing the weight of milled EXTs over the respective volume. Using following equation (Equation (1)), the Carr’s compressibility index (*CI*) was calculated:

(1) $$CI=\;\left(\frac{\rho \;tap-\rho \;bulk}{\rho \;tap}\right)\;\times \;100$$

The Hausner ratio (HR) was obtained by dividing (ρ tap) by (ρ bulk).

### 2.9. Tablet Processing

The milled EXTs were mixed with the tableting excipients of MCC and magnesium stearate (Table 5 and Table 6) and compressed into tablets equivalent to 23 mg of DH with compaction forces of 600 and 1500 psi, respectively. Tablets were compressed on a manual tablet press using a 10 mm round concave punch to a final tablet weight of 300 mg. The tablets, having luminous surfaces, were stored in a plastic bottle until further use. Tablets were evaluated for thickness, hardness, friability, and in vitro release profiles. In addition, drug content and tablet content uniformity tests were also conducted. The DH content was analyzed using the HPLC-UV method described in above section. For improving the drug content, the pre-mixed MCC formulations were compressed on single punch tablet press (MCTMI, Globe pharma, New Brunswick, NJ, USA) using an 8 mm round flat punch with a compaction force of 1500 psi. Magnesium stearate (1%) was added as a lubricant just before the direct compression process, and each compressed tablet weighed 200 mg to mimic the weight of Aricept^®^, which is the commercial product of DH. Aricept^®^ was used as a control formulation in this study.

### 2.10. Tablet Characterization

Some standard characterization tests were performed on tablets manufactured from extruded materials. The thickness and diameter of the tablets were measured using a micrometer. Disintegration testing was performed via a disintegration apparatus. Friability testing was performed in a friabilator (Roche Friabilator, Basel, Switzerland). To test the hardness of the tablets, a VarianVK200 (Agilent technologies, 13000 Weston Pkwy, Cary, NC, USA) hardness tester was used. A tablet content uniformity test was achieved by a drug assay via the HPLC method. The tablet weight uniformity test was performed by measuring each tablet after direct compression via the Mettler Toledo scale (Columbus, OH, USA). Six replicates were tested (*n* = 6).

### 2.11. In Vitro Release Studies

In vitro release studies for either milled EXTs or tablet equivalent to 23 mg of DH were performed using a USP type-II dissolution apparatus with 900 mL of a pH 6.8 phosphate buffer (simulated intestinal fluid) at 37 ± 0.5 °C and 50 rpm for 10 h [18,19]. Due to the extensive use of simulated intestinal fluid for the drug release study of oral and sustained release formulations, it was used as the biorelevant dissolution media for the drug release study in this work [18,19,20]. All dissolution samples were analyzed using an HPLC-UV system (*n* = 3). The paddle speed was set at 50 rpm. The samples were collected at every 0.5, 1, 2, 3, 5, 8, and 10 h, filtered, and then analyzed using an HPLC-UV method. The withdrawn amount for the sample was compensated for by adding an equal amount of drug-free fresh dissolution media back to the dissolution vessel at each time point. The release profile for percent release was plotted against time for each formulation. The model-independent similarity factor f_2_ was used to compare the dissolution profiles among different formulations. In addition, the statistical analysis was conducted using ANOVA to compare the release profiles. It is well-known that if the calculated f_2_ value is between 50 and 100, then the profiles are considered to be similar [20]. In addition, the drug release kinetics of different EXTs were evaluated using different kinetic models such as a zero order model, first order model, the Higuchi model, the Hixson–Crowell model, and the Korsmeyer–Peppas model, as described in the literature [21].

### 2.12. Statistical Analysis

The data were expressed as the mean ± standard deviation (SD). The confidence interval was chosen to be 95%, and a statistically significant difference was determined at a minimal level of significance of 0.05 using Student’s *t*-test.

## 3. Results and Discussion

### 3.1. HME Processing Method and Carriers Screening Studies

The formulations with the lipid were extrudable at the lowest processing temperatures (68–74 °C) (Table 1), whereas E-RS was processed at a relatively higher temperature (150 °C), as described in Table 3. A combination of DH with a mixture of the lipid and EC-N7 (1:1:1) led to a relatively lower processing temperature (100 °C) compared to that of DH with EC-N7 (140 °C), as described in Table 2. Stearic acid was utilized as a processing aid. The formulation was compressed and densified in a metering section and die. In our preliminary study, we processed some batches through manual feeding and noticed that when we stopped the feeding, there was more residence time and less pushing force caused by the solid lipid material. This caused the lipid to be melted, decreased the output product, and increased the processing time. To identify a suitable carrier matrix to produce an ideal and stable release profile that conformed to USP specifications and mimicked the marketed formulation (Aricept^®^), different carriers such as EC-N7, E-RS, and C-888 were evaluated for extrudability and desired release profiles.

The optimized HME processing conditions for carriers’ screening are shown in Table 1, Table 2 and Table 3. Regarding the stable sustained release profile, we found out that the lipid was the most appropriate for drug release profiles. It gave us a nearly ideal zero order release profile. It showed no burst release compared to other studied carriers. When EC-N7 and E-RS were used as the matrix-forming agents, DH release was faster within the first hour; this was not preferable and could give spikes in plasma drug levels. The lipid was selected for further investigations since it did not give an initial burst release and retained the crystallinity of DH. In addition, in the HME process, there was DH transformation into its partial or total metastable amorphous form, as in the case of E-RS. DH could recrystallize during storage and, as a consequence, affect the dissolution profile, which was important given that our goal in this study was to keep a stable sustained release formulation. To make sure that we preserved the drug’s crystallinity, we preferred to process at the lowest processing condition. A lipid can be used as a processing aid, and it is an alternative to a chemical plasticizer [22]. Here, the lipid did not undergo to any chemical degradation such as acid hydrolysis when extruding at higher temperature [23]. In the case of extruding the lipid at a higher screw speed, the lipid started to be liquefied due to the friction of the screw generating an extra source of heat. Therefore, we chose a 50 rpm screw speed as the optimized speed instead of 100 rpm. A lower-than-50-rpm speed causes more residence time and liquefies the lipid, and there is a risk of the degradation of the API because of longer residence time attained. When the lipid was extruded above the melting point, the resulting material was in the liquid form. However, when the lipid was extruded below its melting point, it gave granular/powdery solid rod EXTs. Extruding with EC-N7 and E-RS at the set temperature gave opaque solid rod EXTs. C-888, due to its thermostable nature, was utilized to form HME matrices. C-888 melted at around 74 °C and extruded below its melting point at around 70 °C [4,24,25,26]. There are several advantages of using C-888 as a processing aid instead of chemical plasticizers and other hydrophobic polymers. One of these advantages is the lower processing torque compared to EC-N7 and E-RS alone that can be attained due to lower melting point of C-888. When we used C-888 as a processing aid, the processing temperature was significantly decreased to 100 °C for EC-N7. The barrel temperature and screw speeds of the hot-melt extruder were optimized for maximum yield and were set according to observations made from thermal studies and the final EXT. Therefore, all three hydrophobic-forming matrices with DH showed an excellent extrudability under the utilized processing parameters. The blends of the lipid and hydrophobic polymers could be extruded and result in improved physico-mechanical properties; however, the process needs further optimization in terms of dissolution profiles, which is the subject of future studies.

### 3.2. Tablet’s Excipient MCC Co-Processed with Lipid to Improve Tablet Integrity

The co-extrusion of premixed tablet excipients is a critical aspect in the pharmaceutical industry that has yet to be completely addressed. One of the major challenges while extruding lipids is that they have a poor flowability within an extruder barrel. This caused drug content and content uniformity variations in this research. For optimized lipid formulation besides EC-N7 and E-RS, the C-888–DH–MCC interaction, DH crystallinity, dissolution behavior, tablet integrity, and lipid aging/API stability studies are critical aspects in this work that should be themes of future studies. A lipid can be extruded with tablet excipients such as dibasic calcium phosphate anhydrous (DCPA) [12,25]. To the best of our knowledge, MCC was used here for the first time to extrude as a tablet’s excipient. The co-processing of tablet’s excipients is assumed to reduce processing steps (i.e., the blending of tablet excipients), labor work, and manufacturing time. Therefore, premixed tablet robustness in terms of drug content, tablet content uniformity, hardness, friability, and dissolution profile were investigated in our study. Tablet compositions are shown in Table 5 and Table 6.

**Figure 1:**
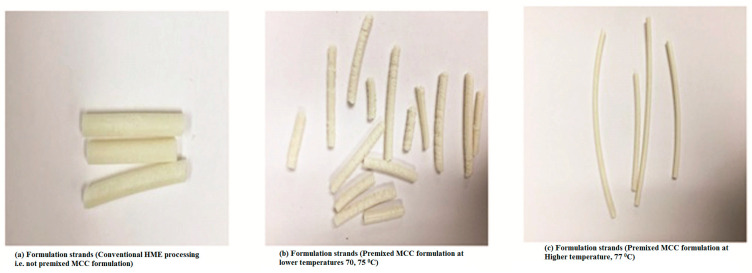
Formulation strand digital pictures.

The extruding temperatures zones for the premixed MCC formulations were optimized and set to get continuous and neat rod EXTs (Figure 1) at temperatures above the melting point of C-888 for the optimized formulations for further investigations. MCC was added to lipid matrix because of the poor cohesive forces between the lipid particles since a lipid is extremely hydrophobic in nature [12]. Therefore, the co-processing premixed MCC approach was utilized to improve the tablet integrity. The torque for the premixed MCC formulations compared to DH with the lipid alone was higher because there were more solid-state particles of DH and MCC. MCC had the high melting point of around 270 °C, and it increased the resistance of the barrel because of frication forces that led to a higher torque. For the optimized formulation after screening stage, when processing MCC for conventional tablet preparations, solid bridges can be created by mechanical force via direct compression. The MCC is not completely impeded within tablet matrix. However, interconnection bond after melting the lipid [27,28,29] and intragranular and intergranular bonds within the lipid while extruding the blends might occur [30]. Therefore, it can be concluded that this fusion bonding of MCC with the lipid while extruding made the MCC completely embedded within the matrix and prevented tablet splitting and burst release in the dissolution media, instead leading to the complete disintegration that occurred after 2 h in our case when MCC was not co-processed via HME with the rest of formulation components. For the formulation of a 300 mg tablet of DH with the lipid, the separation of the tablet into parts was the main issue after contact with dissolution media after 15 min (i.e., cracking and capping), thus leading to a faster release and a complete release within 5 h. Therefore, we proposed the co-extrusion of the tablet excipient MCC to observe its effect on tablet integrity.

The measured hardness with the co-extruded material was independent to the compaction force, although the co-extruded formulation was mixed with a filler (i.e., MCC). However, with conventional methods of adding tablet excipient, the hardness was dependent on the compaction force. This can be explained as the MCC being embedded deeply within the matrix in the case of premixed formulations, whereas, in the conventional formulation, MCC particles surrounded the solid dispersion particles of DH and the lipid. The friability was less 1%, indicating lipid matrix tablet robustness. The EXT granules showed a good *CI* that was between 15 and 16%. The smaller Carr’s compressibility index was because of the hydrophobic nature of lipids. After co-extrusion with these kinds of fillers, there was no capping and lamination when in contact with the dissolution media. In addition, there was no lipid melting, egress, or spread during tablet compression, which is what we faced with conventional tablet processing in the case of a high ratio of the lipid. These findings were in agreement with a previous study [30]. The formulations with C-888 and MCC were extrudable at the lowest processing temperatures (70–75 °C), but the strands were defective (Figure 1b). However, they were harder and neater at higher temperature of 77 °C (Figure 1c) compared to the DH:lipid tablets (Figure 1a), which were difficult to handle. These harder strands would be beneficial in downstream processing. The tablet formulation consisting of DH:lipid:mannitol (1:2:0.075)-milled EXTs demonstrated an average tablet hardness of 10 kp and a thickness of 4.24 mm. The tablets were less than 0.1% friable, but a faster release and capping issues in dissolution media were observed. Therefore, we utilized the co-extrusion of the tablet excipients. The premixed formulation of milled EXT tablets demonstrated an average tablet hardness of 6.8 kp and a thickness of 3.2 mm. In addition, the friability was less than 1%. The hardness decreased because of the decrease of the tablet weight (i.e., to 200 mg to mimic the marketed formulation), which led to the amount of MCC being decreased. MCC has been reported to increase tablet strength at a certain amount and ratio. The Hausner ratio for the premixed formulation was 1.13, which indicated good flow properties.

### 3.3. Heat Fusion Processing Method

The objectives of this study were to investigate the influence of traditional heat fusion processing techniques on lipid-based formulations and compare them to those prepared by the HME processing techniques. The fusion method produced granules from a congealed mass. This congealed mass has been reported to decrease the drug release behavior compared to the traditional direct compression and wet granulation methods. The efficient and intense mixing while extruding the material inside the barrel was able incorporate more API particles into the lipid matrix and make a formulation with a slower release behavior compared to heat fusion (data not shown). The reason for this might be that the pore architecture was different in the case of the formulation prepared using continuous HME. The structure had less porosity compared to formulation prepared by batch process heat fusion. The HME process gave us a more stable and slow release compare to heat fusion. Formulations prepared by heat fusion gave a burst release. Our results were in agreement with those of a previous research group [3]. The stirring force was not as efficient as shearing force by HME, where DH could be completely embedded within the matrix, which was the same behavior as that of tramadol HCl prepared by the heat fusion method that gave a burst release; in our case with HME, this did not occur because of the complete embedding via shearing force [3].

### 3.4. TGA Analysis

Prior to HME, we could determine the maximum processing temperatures by utilizing TGA. This analysis indicated the thermal stability for APIs and excipients/carriers being processed via HME by determining the amount of weight loss due to decomposition as a function of temperature. In present study, it was found that less than 2% of degradation below 250 °C for the neat materials, physical mixtures, and EXTs at various drug:carrier ratios and different temperature profiles was observed (data not shown). TGA confirmed the thermal stability of API and excipients/carriers at the extrusion temperature utilized during processing, as well as providing a working temperature range. Therefore, as a crucial aspect, it was concluded in our study that the studied processing temperatures were convenient to employ during the extrusion process.

### 3.5. DSC Analysis

**Figure 2:**
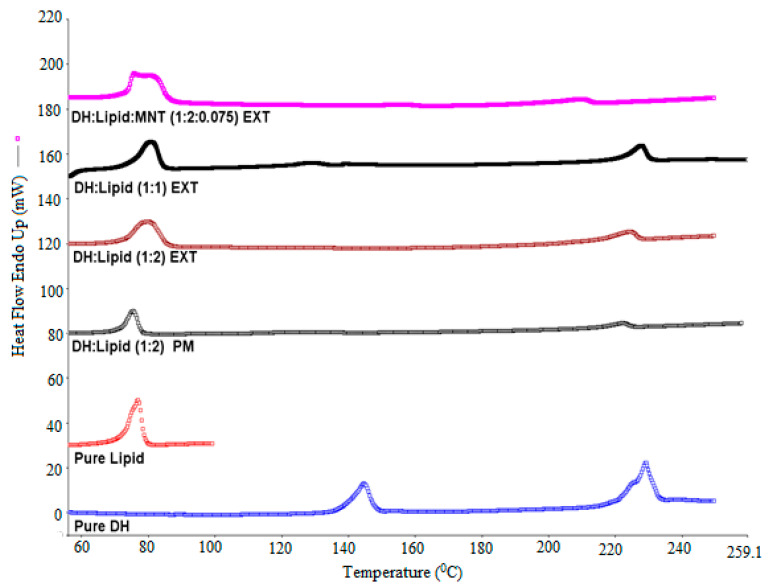
DH/C-888 and DH/C-888/MNT DSC studies performed at temperatures of 30–250 °C and a heating rate of 20 °C/min.

**Figure 3:**
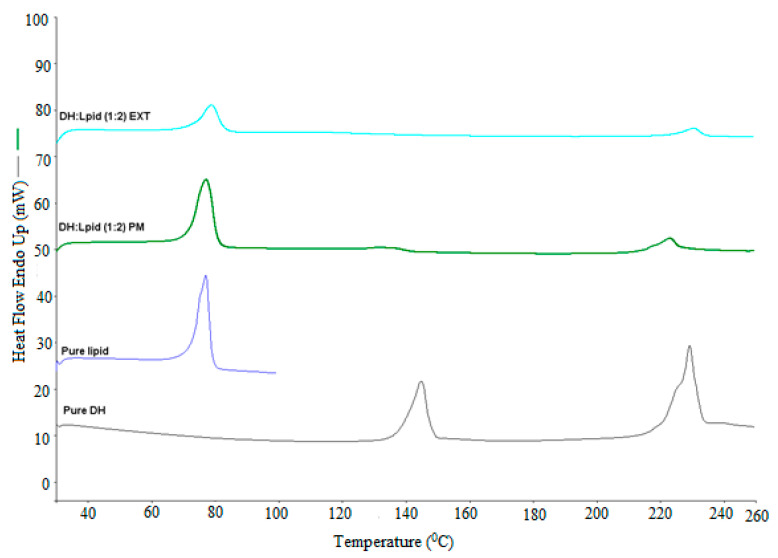
DH/C-888 DSC studies performed at temperatures of 30–250 °C and a heating rate of 20 °C/min.

**Figure 4:**
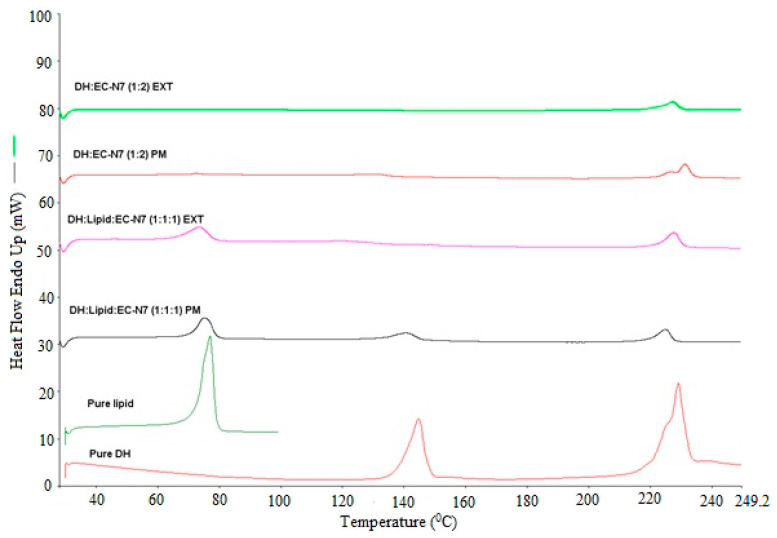
DH/C-888/EC-N7(1:1:1) and DH/EC-N7(1:2) DSC studies performed at temperatures of 30–250 °C and a heating rate of 20 °C/min.

**Figure 5:**
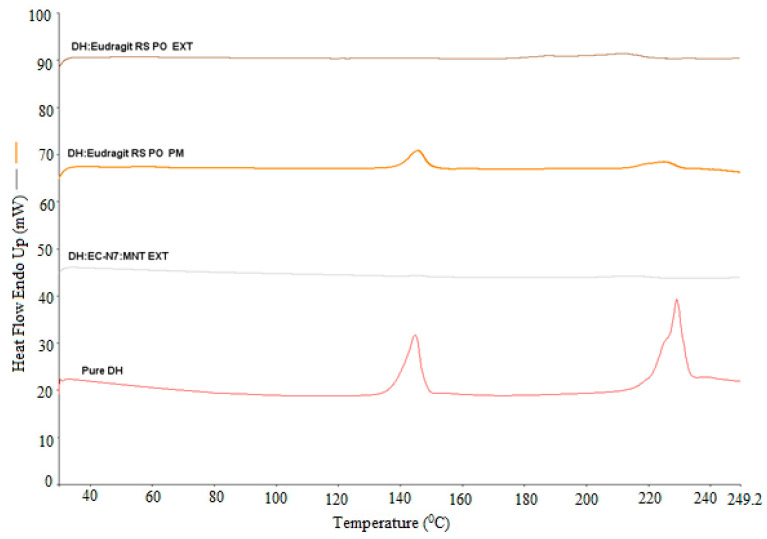
DH/Eudragit^®^ RS PO (1:2) and DH/EC-N7/MNT (1:2:0.1) DSC studies performed at temperatures of 30–250 °C and a heating rate of 20 °C/min.

The solid dispersion EXTs is generally characterized by thermal and diffraction techniques such as DSC and X-ray diffraction to determine the API state within hydrophobic matrix. The physical state of DH was found to be either crystalline or amorphous depending on carrier and processing conditions used. In this study, DSC was utilized to characterize the physicochemical properties of physical mixture (PM), solid dispersions (EXT), and individual neat components. Since the change in crystallinity/amorphous state or change from one polymorph to another often exhibits significant differences in solubility, bioavailability, processability, and physical/chemical stability, these thermal studies were conducted to evaluate the effect of processing on the crystallinity of the DH and the lipid. As we can see in the DSC thermograms (Figure 2, Figure 3, Figure 4 and Figure 5), neat DH showed two characteristic melting endothermic peaks at around 145 and 228 °C [31]. In addition, the lipid showed a characteristic melting endothermic peak at around 74 °C. For all EXTs, the first melting endothermic peak completely disappeared. The EXTs containing E-RS as a hydrophobic carrier or MNT as a pore-former did not exhibit melting endotherms, thus indicating that DH was rendered amorphous during the extrusion process, which was not preferable in our study since the DH is already a highly soluble drug. The melting endotherm for the lipid was apparent in all EXTs but less intense (i.e., less delta H “enthalpy”) compared to the pure lipid and physical mixture, thus indicating that the extent of crystallinity of the lipid was greatly reduced. This means that extrusion process had an effect on lipid crystallinity, and this was beyond the scope of this study. Lipid endothermic melting peaks were slightly shifted to around 70 °C for all drug loadings, thus indicating negligible drug–lipid interactions. Regarding the formulation that had a mixture of EC-N7 and the lipid (1:1) and contained DH, it was found that the lipid was dispersed within the EC-N7. The reduced melting endotherm of lipid in EC-N7:lipid formulation showed that a fraction of the lipid material recrystallized as a separate phase before EC-N7 vitrified (solidified) [32]. This crystalline phase of the lipid was observed in the DSC thermograms. These findings mean that in some formulations, DH had a partial crystallization within the carrier matrix and had not completely converted to a higher energy amorphous form. This amorphous form was not preferable in our study because of the risk of related stability issues. Such an issue would consequently affect the relevant dissolution profile and other physicochemical aspects.

### 3.6. SEM Analysis for Carriers’ Screening

**Figure 6:**
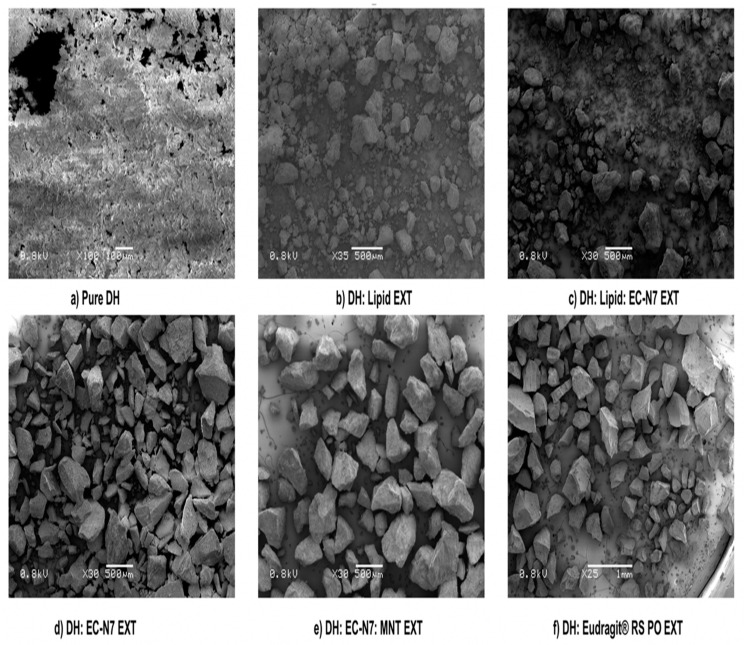
SEM of pure DH and screened extrudates.

The surface morphology was examined by SEM for all EXTs. As can be seen in Figure 6, the pure drug crystals appeared to be irregular in shape and size as aggregates of the needle form, i.e., microcrystalline aggregates with a coarse surface. The DH:lipid (1:2), DH:lipid:EC-N7 (1:1:1), and DH:EC-N7 (1:2) formulations showed the presence of DH in the microcrystalline aggregate needle form, which was distinguishable from other formulations. In the case of EXTs of the formulations of DH:EC-N7:MNT (1:2:0.1) and DH:E-RS PO (1:2), it was observed that the aggregates disappeared, which signified the presence of an amorphous DH within the hydrophobic matrices during the extrusion processing, as confirmed by DSC results. In other words, no aggregation was noticed, thus suggesting a completely miscible, single-phase, binary solid dispersion system. SEM demonstrated that the process enabled active ingredients to be embedded into an insoluble matrix since there were no any crystals on the surfaces—only smooth surfaces [33].

### 3.7. Solubility Parameter Calculation for Optimized Formulation

The three-dimensional Hansen solubility parameter imparts information about the physical state and interactions such as the dispersive, polar, and hydrogen bonding interactions of a molecule, and it could be used to characterize the interactions between formulation’s compositions [34]. It is very useful tool in pharmaceutical applications. It could predict API morphology, drug-carrier miscibility in the solid dispersions, and drug–excipient interactions in the field of pharmaceutical development. The effective solubility parameters obtained from Hansen method for DH and lipid were 28.3 and 19.6 MPa^1/2^, respectively. Theoretically, in order to observe an at least partial miscibility between the drug and the carrier, the solubility parameter difference (Δδ) between them is required to be less than 7.0 MPa^1/2^ [35,36]. In the present study, (Δδ) was 8.7, which was significantly higher than 7.0 MPa^1/2^, thus indicating the immiscibility of DH with the lipid.

### 3.8. Drug Content and Content Uniformity

The conventionally-processed tablets showed variations in drug content and content uniformity because of inefficient drug flow inside the barrel due to the low melt viscosity of the lipid. Such variations make the extrusion of lipids a challenging process [37]. The drug content and tablet content uniformity were improved when extruding the formulation with MCC. Drug content and tablet content uniformity analyses displayed that the drug was uniformly distributed within the tablets with a standard deviation of <4%, thus indicating a good formulation and process. The reason behind the improvements of drug content and content uniformity was that the MCC played a vital role in improving lipid flowability because it enhanced the drug flow and consistency of mixing inside the extruder barrel. Therefore, there were good dispersive and distributive effects of the DH particles inside the lipid matrix. The premixed MCC formulation exhibited a 102.4% drug content compared to those without MCC, which was found to be approximately 71.4% (*p* < 0.05) using an unpaired *t*-test.

### 3.9. Mechanism of MCC Behavior on Drug Release from Tablet Prepared by Conventional Tableting Method (i.e., Not Co-Processed MCC Formulation)

MCC adsorbed water into the tablet through capillaries, leading to the swelling and eventual disintegration of the tablets. New surfaces were thus created for drug diffusion to occur. This finding was in agreement with that of El-Shanawany, who reported a faster release of nitrofurantoin from lipid tablets containing MCC [38].

### 3.10. In Vitro Release Study for the Screened Milled Extrudates Formulations

The lipid gave a lubrication effect to EC-N7 to decrease the processing temperature but could not solve the burst release behavior of EC-N7. The release kinetic parameters of different extrudates were evaluated using different mathematical models, and the results are summarized in Table 7.

**Figure 7:**
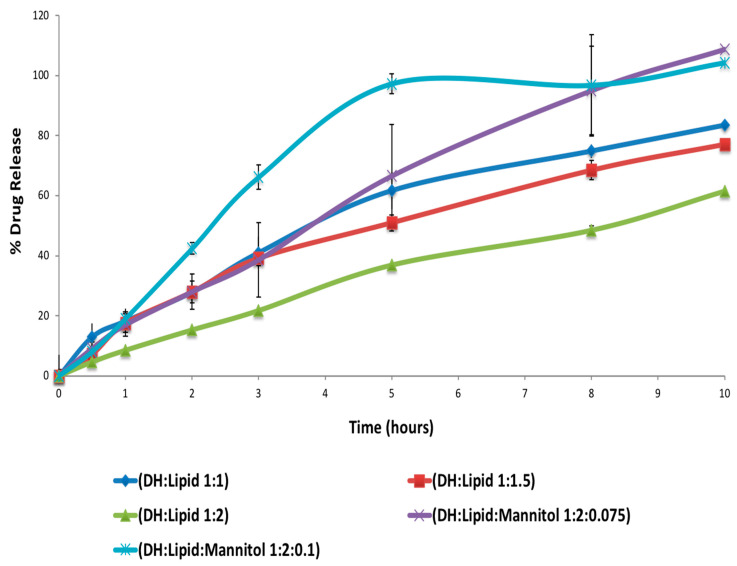
In vitro drug release of DH (mean ± SD; *n* = 3).

**Figure 8:**
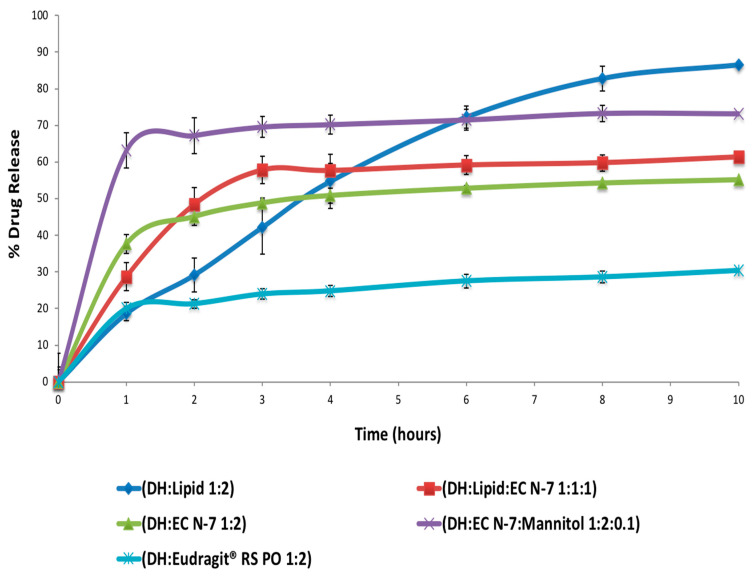
In vitro drug release of DH from different formulations (mean ± SD; *n* = 3).

As shown in Figure 7, the formulation of DH:lipid:mannitol (1:2:0.075) demonstrated sustained, stable, and complete release in 10 h. Dissolution test showed various release profiles, as seen in Figure 8, according to different carriers’ characteristics. The addition of MNT enhanced the drug release due to its pore-forming capability. The pore formation by MNT could have reduced the particle size of the extrudates, which could have resulted in an enhanced surface area for drug dissolution/absorption. Hence, the enhanced release of DH from formulations containing MNT was possible due to particle size reduction via pore formation by MNT. Using only the lipid as a carrier in the formulation resulted in a nearly ideal (zero-order) release profile, as indicated by the values of determination coefficients (R^2^) summarized in Table 7. The R^2^ values for these formulations were found to be maximum for the zero order kinetic model compared to other studied models. These formulations (with the lipid only) followed non-Fickian diffusion mechanism, as the diffusion exponent (n) for these formulations was less than 1.0 but greater than 0.5 [21]. However, the physical properties of these extruded material were not suitable for further processing, such as tableting or capsule filling operations (i.e., in our case, sticking in capsules, tablet separation, and tablet capping while in dissolution, which resulted in a faster burst release). The EXTs containing the lipid and MNT followed the Peppas model of drug release with a non-Fickian diffusion mechanism. The other studied EXTs followed the Peppas model of drug release with a Fickian diffusion mechanism as the n values were less than 0.5 for such formulations [21].

The film coating technique is a conventional technique to prevent burst release, but in the case of a lipid, there is no need to perform it. Burst release phenomena could be solved by film coating, but we solved the burst release with HME lipid extrusion. The initial burst release from the matrix could probably be attributed to the dissolution of the drug from the surface of the milled EXTs. This issue could be solved via HME but only in the case of a lipid, not E-RS or EC-N7. The goal of adding lipid in screening of polymers was to decrease the extent of initial burst release compared to other formulations, as shown in Figure 8. It was also done to avoid dose dumping caused by food agitation with other gel forming matrices [3,39]. The reason why E-RS provided a more sustained release was because it is a non-permeable polymer. Furthermore, we excluded E-RS and EC-N7 because they did not provide consistent release profile and gave a burst release during the first few hours. This observation was also supported by the release kinetics data, as no consistent R^2^ values were recorded for these formulations. In the last few hours, the release profile was slightly increased, because it was below the percolation threshold [40], thus leading to incomplete drug release. This phenomenon usually happened due to the limited access of deeper drug particles to the dissolution media since they were completely embedded in the hydrophobic EC-N7 and E-RS. During the initial stage of the dissolution test, the drug clusters located on the surface of the granules were readily released into the dissolution medium. As the dissolution process progressed, fewer drug clusters were accessible to the dissolution medium, and a plateau was reached [29]. This plateau behavior was because the mixture was under the percolation threshold. Below this threshold, incomplete drug release is observed, presumably due to the limited accessibility of many drug particles to the dissolution medium since they are encapsulated by water-insoluble polymeric materials [29,40].

### 3.11. Effect of Varying Processing Temperature and Lipid Ratio on Dissolution Profile of Milled EXTs

**Figure 9:**
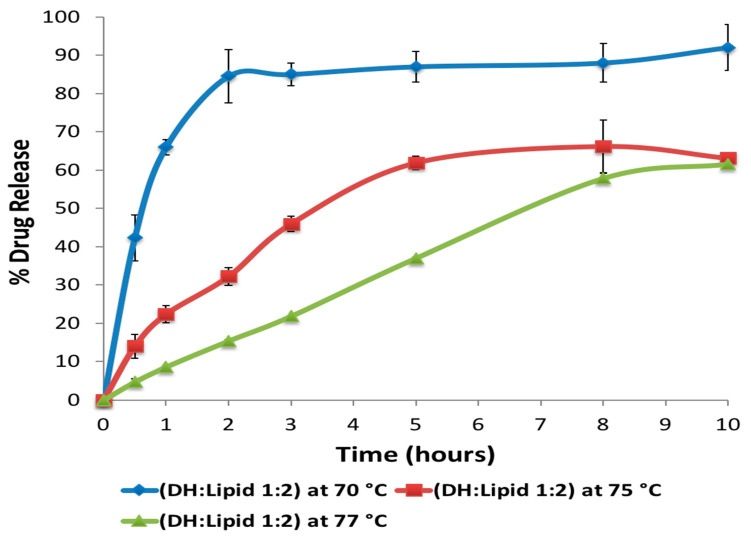
Effect of HME processing temperatures on the in vitro release profile of DH (DH: Lipid 1:2) (mean ± SD; *n* = 3).

The effect of extrusion temperature and C-888 ratio on the release profile was studied. It has been confirmed in previous studies that the processing temperature plays a significant role in the tortuosity of a carrier [40,41,42,43]. At the processing temperature of the melting point of the lipid and above (i.e., 74 °C and above), formulations processed via HME did not give any fast release because of particles being on surface at the beginning, which meant that because of the shearing force and mixing, the distributive and dispersive mixing played roles in completely embedding the DH particles within the lipid matrix (Figure 9).

### 3.12. Dissolution Studies of Processed Tablets

MCC works by swelling in water and, eventually, disintegrating the matrix, like what happened in our conventional tablets. The release from all the prepared tablets was studied under different pH values. It was observed that the release of DH from such matrices was independent of the pH since the release rates were very similar under the studied pH values. This result was expected because DH is the salt of a basic drug with a pKa of 8.2. Therefore, it was ionized over the pH range of interest and soluble at all pH values used in this study, and it can be concluded that the matrix was inert towards changes in the pH. The presence of MCC, which is a hydrophilic polymer, is not preferable for water-soluble APIs, but this could be solved by premixed co-extrusion. A faster and less consistent release was achieved from the conventionally prepared tablets comprising MCC, and the structures were observed to swell and laterally split into layers during dissolution testing, which was ascribed to the nature of MCC that promoted disintegration [44]. The addition of MNT enhanced the drug release due to its pore-forming capability. The formulation of DH:lipid:MNT (1:2:0.075) demonstrated a sustained, stable, and complete release in 10 h.

**Figure 10:**
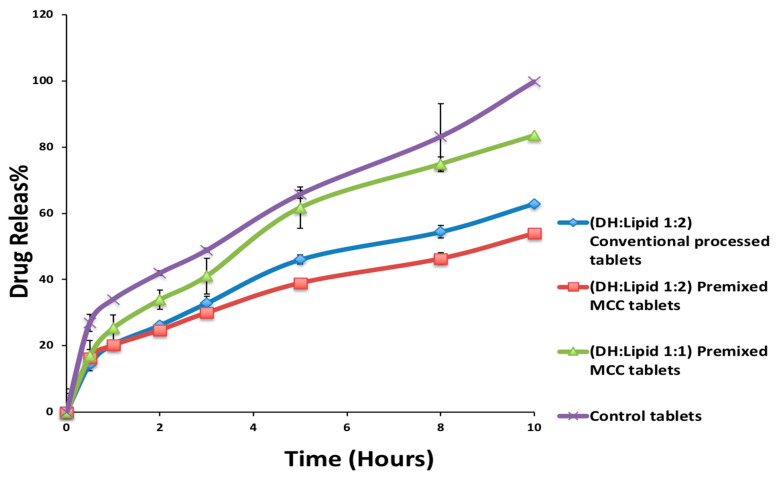
In vitro drug release profile of DH from optimized DH tablets and marketed product (control) (mean ± SD; *n* = 3).

The premixed MCC formulation containing a mixture of DH, C-888 ATO (1:2), and MCC exhibited a more sustained drug release compared to the marketed Aricept^®^ formulation. However, after increasing the drug ratio to (1:1), the drug release was enhanced and reached up to 81% after 10 h (Figure 10) compared to control tablets (100% after 10 h). After 10 h in the dissolution media, the premixed MCC tablets remained intact with no cracks, and this explained the lower drug release profile compared to the marketed Aricept^®^. The higher release profile of DH from the marketed Aricept^®^ formulation compared to optimized formulations was due to the fact that marketed tablets utilized a film coating to extend the release of DH compared to optimized formulations. In addition, the conventionally processed tablets had cracks in their middles. This explained the faster release profile. However, in the formulation of MNT (0.1%), there was a burst release. The *f*_2_-similarity factor between DH:lipid (1:1) and the control tablet was calculated as 54 (more than 50 means similar profiles).

## 4. Conclusions

DH can be formulated in a solvent-free, one-step, cost-effective, and continuous process using C-888 as a sustained release matrix with MNT as a hydrophilic pore-former. It was confirmed that DH in a lipid matrix was immiscible, and it preserved its crystallinity during the extrusion process. However, some fractions of DH were solubilized in the lipid matrix with no endothermic peaks, whereas the formulations with MNT were not able to maintain DH crystallinity. The optimized formulation (DH:lipid:mannitol; 1:2:0.075) demonstrated a sustained, stable, and complete release in 10 h. In vivo investigations are required to correlate with in vitro dissolution studies for the development of optimum oral sustained release matrix tablets of DH, which is the scope of a future study. The prepared tablets showed good post-compression characteristics regarding hardness, friability, weight variation, content uniformity, and desired and reproducible drug release profiles compared to the reference commercial product. It was concluded from the dissolution profiles that the HME technique can be utilized for the manufacturing of DH sustained release tablets via the co-processing of tablet excipients with API/C-888. Tailoring the ratio of C-888 and MNT in the formulations, along with an appropriate extrusion temperature profile, resulted in a modification of the release of DH and the obtainment of a preferable release pattern.

The co-extrusion of tablet excipient (MCC) with an API/matrix-former could be a problem-solving approach for the development of lipid-based hydrophilic APIs. This could contribute to a reduction of processing steps, such as blending, as well as improving the integrity of lipid-based tablets. The HME processing temperature profile plays key factor in the dissolution behavior of DH. It was demonstrated that the co-extrusion of tablet excipients within an API/lipid matrix resulted in different dissolution behaviors and tablet properties. Though DH was in the amorphous form in the case of the formulation that contained hydrophilic diluents/fillers such as MNT and MCC, the release was sustained and stable. The only explanation is that these hydrophilic excipients were embedded within the C-888 matrix. This new insight shows a new trend in terms of preparing a developed and continuous tableting process (e.g., mini matrices) in the solid lipid extrusion field. This insight could give useful information in terms of product development.

## Figures and Tables

**Table 1:** Formulation compositions and processing parameters for lipid carrier. DH: donepezil hydrochloride; C-888: Compritol^®^ 888 ATO; MNT: mannitol.

DH (Ratio *w*/*w*)	C-888 (Ratio *w*/*w*)	MNT (Ratio *w*/*w*)	Zone/Temp.	Screw Speed
1	4	N/A	Zone 2–3–4: 68 °C	50/100 rpm
1	3	N/A	Zone 5–6–7: 74 °C	
1 *	2 *	N/A or 0.1 or 0.075	Zone 8–9–10 and die: 68 °C	
1	1	N/A		

* (1:2) (DH:C-888): Additionally, processed by conventional heat fusion method at processing temperature 100 °C.

**Table 2:** Formulation compositions and processing parameters for ethyl cellulose N7 (EC-N7) with/without a lipid carrier.

DH (Ratio *w*/*w*)	EC N-7 (Ratio *w*/*w*)	C-888 (Ratio *w*/*w*)	MNT (Ratio *w*/*w*)	Stearic Acid (Ratio *w*/*w*)	Zone/Temp.	Screw Speed
1	4	N/A	N/A	0.05	All zones: 140 °C	100 rpm
1	3	N/A	N/A	0.05		
1	2	N/A	0.1	0.05		
1	1	N/A	N/A	0.05		
1	1	1	N/A	N/A	All zones: 100 °C	

**Table 3:** Formulation compositions and processing parameters for the Eudragit^®^ RS PO (E-RS) carrier.

DH (Ratio *w*/*w*)	Eudragit^®^ RS PO (Ratio *w*/*w*)	Zone/Temp.	Screw Speed
1	4	All zones: 150 °C	100 rpm
1	3		
1	2		
1	1		

**Table 4:** Hot-melt extrusion (HME) processing conditions to study the effect of HME processing temperature on lipid-based formulations and drug release profiles.

HME Parameters	Processing Conditions
Barrel Temperature Profiles (°C)	Zone 2–7: 77 °C, Zone 8–10 and Die: 70 °C
	Zone 2–7: 75 °C, Zone 8–10 and Die: 70 °C
	All zones: 70 °C
Screw Speed (rpm)	50 rpm

**Table 5:** Tablet compositions for conventionally processed, 300 mg tablets. MCC: microcrystalline cellulose; EXT: extrudate.

Material	33.33% DH/C-888 EXTs		50% DH/C-888 EXTs	
	% (*w*/*w*)	Weight (mg/Tablet)	% (*w*/*w*)	Weight (mg/Tablet)
DH	8	23	7.67	23
C-888	16	46	7.67	23
Avicel^®^ PH 102 (MCC)	75	228	83.66	251
Magnesium Stearate	1	3	1	3
Total	100	300	100	300

**Table 6:** Tablet compositions for conventionally processed, 200 mg tablets.

Material	33.33% DH/C-888 EXTs		50% DH/C-888 EXTs	
	% (*w*/*w*)	Weight (mg/Tablet)	% (*w*/*w*)	Weight (mg/Tablet)
DH	11.5	23	11.5	23
C-888	23	46	11.5	23
Avicel^®^ PH 102 (MCC) *	64.5	129	76	152
Magnesium Stearate	1	2	1	2
Total	100	200	100	200

* Additionally co-processed while extruding the formulation.

**Table 7:** Drug release kinetic parameters for drug release of DH from different extrudates.

Formulation	Zero Order		First Order		Higuchi	Hixson–Crowell	Peppas	
	K_0_	R^2^	k_1_	R^2^	R^2^	R^2^	R^2^	*n*
DH:lipid (1:2)	7.71	0.9930	1.54	0.9872	0.9864	0.9857	0.9632	0.721
DH:lipid (1:1.5)	7.52	0.9815	1.38	0.9321	0.9314	0.9241	0.9521	0.701
DH:lipid (1:1)	7.41	0.9710	1.33	0.9202	0.9141	0.9032	0.9610	0.645
DH:EC-N7 (1:2)	1.64	0.73976	1.07	0.7794	0.8527	0.7665	0.9198	0.165
DH:E-RS (1:2)	1.38	0.8924	1.03	0.9648	0.9889	0.9627	0.9791	0.187
DH:lipid:EC-N7 (1:1:1)	2.13	0.5798	1.10	0.6252	0.7097	0.6099	0.8083	0.215
DH:lipid:MNT (1:2:0.1)	6.24	0.9680	1.41	0.8521	0.8412	0.8089	0.9780	0.715
DH:lipid:MNT (1:2:0.075)	4.22	0.9260	1.06	0.8112	0.7821	0.7011	0.9411	0.516
DH:EC-N7:MNT (1:2:0.1)	1.13	0.9581	1.11	0.8580	0.9032	0.8412	0.9619	0.117

R^2^: determination coefficient; K_0_: zero order rate constant; k_1_: first order rate constant; and n: diffusion exponent.

## Data Availability

Not applicable.
